# Low-Intensity mental health Support via a Telehealth Enabled Network for adults with diabetes (LISTEN): protocol for a hybrid type 1 effectiveness implementation trial

**DOI:** 10.1186/s13063-023-07338-5

**Published:** 2023-05-23

**Authors:** Edith E. Holloway, Shikha Gray, Cathrine Mihalopoulos, Vincent L. Versace, Roslyn Le Gautier, Mary Lou Chatterton, Virginia Hagger, Jennifer Halliday, Kim Henshaw, Benjamin Harrap, Sarah Manallack, Taryn Black, Natasha Van Bruggen, Carolyn Hines, Adrienne O’Neil, Timothy C. Skinner, Jane Speight, Christel Hendrieckx

**Affiliations:** 1 School of Psychology, Deakin University, Geelong, VIC Australia; 2 The Australian Centre for Behavioural Research in Diabetes, Diabetes Victoria, Melbourne, VIC Australia; 3 Institute for Health Transformation, Deakin University, Geelong, Australia; 4 School of Public Health and Preventive Medicine, Monash University, Melbourne, VIC Australia; 5 School of Medicine, Deakin Rural Health, Deakin University, Warrnambool, VIC Australia; 6 School of Nursing & Midwifery, Deakin University, Burwood, VIC Australia; 7 Victoria, Australia; 8 Diabetes Australia, Milton, QLD Australia; 9 Diabetes Victoria, Victoria, Australia; 10 IMPACT Institute, Deakin University, Geelong, VIC Australia; 11 Department of Psychology, La Trobe University, Victoria, Australia; 12 Department of Psychology, University of Copenhagen, Copenhagen, Denmark

**Keywords:** Diabetes, Diabetes distress, Mental health, Emotional support, Diabetes health professionals, Problem-solving, Telehealth

## Abstract

**Background:**

Mental health problems are common among people with diabetes. However, evidence-based strategies for the prevention and early intervention of emotional problems in people with diabetes are lacking. Our aim is to assess the real-world effectiveness, cost-effectiveness, and implementation of a Low-Intensity mental health Support via a Telehealth Enabled Network (LISTEN), facilitated by diabetes health professionals (HPs).

**Methods:**

A hybrid type I effectiveness-implementation trial, including a two-arm parallel randomised controlled trial, alongside mixed methods process evaluation. Recruited primarily via the National Diabetes Services Scheme, Australian adults with diabetes (*N* = 454) will be eligible if they are experiencing elevated diabetes distress. Participants are randomised (1:1 ratio) to LISTEN—a brief, low-intensity mental health support program based on a problem-solving therapy framework and delivered via telehealth (intervention) or usual care (web-based resources about diabetes and emotional health). Data are collected via online assessments at baseline (T0), 8 weeks (T1) and 6 months (T2, primary endpoint) follow-up. The primary outcome is between-group differences in diabetes distress at T2. Secondary outcomes include the immediate (T1) and longer-term (T2) effect of the intervention on psychological distress, general emotional well-being, and coping self-efficacy. A within-trial economic evaluation will be conducted. Implementation outcomes will be assessed using mixed methods, according to the Reach, Effectiveness, Adoption, Implementation, and Maintenance (RE-AIM) framework. Data collection will include qualitative interviews and field notes.

**Discussion:**

It is anticipated that LISTEN will reduce diabetes distress among adults with diabetes. The pragmatic trial results will determine whether LISTEN is effective, cost-effective, and should be implemented at scale. Qualitative findings will be used to refine the intervention and implementation strategies as required.

**Trial registration:**

This trial has been registered with the Australian New Zealand Clinical Trials Registry (ACTRN: ACTRN12622000168752) on 1 February, 2022.

**Supplementary Information:**

The online version contains supplementary material available at 10.1186/s13063-023-07338-5.

## Background

Around 50% of adults with diabetes experience mental health problems [1, 2], including depression, anxiety and diabetes distress (i.e. the negative emotional or affective experience resulting from the challenge of living with the demands of diabetes) [3]. There is increasing recognition of the impact of living with diabetes on emotional and mental health, and that this needs to be addressed as part of comprehensive diabetes care [4, 5]. Mental health problems are a major obstacle to effective diabetes self-management [6] and persistent diabetes distress can be a precursor to depression [7]. Both are associated with sub-optimal glycated haemoglobin (HbA1c: an important indicator of risk for macrovascular and microvascular complications) [8], reduced quality of life, work absenteeism, and increased healthcare costs [9–12]. Early intervention, using evidence-based approaches, may prevent the escalation of symptoms to severe psychological distress, enhance self-management behaviours and improve quality of life, health outcomes [13].

Importantly, people with diabetes want to discuss their emotional well-being with diabetes health professionals (HPs) [14], and they value their diabetes HPs showing empathy and acknowledging the emotional challenges faced in self-managing their condition [15]. However, data from a multi-national survey (17 countries) of diabetes health professionals showed that such discussions tend to be limited [16]. HPs report a lack of training, skills, confidence, time and other resources to attend to the emotional needs of people with diabetes [6], and would like further training and support [16, 17].

There is encouraging evidence for the effectiveness of diabetes-tailored psychological interventions for reducing diabetes distress [18]. However, such interventions are rarely implemented by HPs in clinical practice due to the aforementioned barriers, including a lack of training and skills. Diabetes HPs may be well-placed to deliver low-intensity mental health interventions, which are brief, and aim to provide a less costly approach than ‘standard’ psychological therapy [19]. Such interventions focus on supporting self-management and skills development, typically do not require delivery by a mental health professional, and provide a key service platform within a mental health stepped care model [19]. Brief problem-solving therapy (PST) is an evidence-based, low-intensity psychological intervention in which the person is supported by a health professional to learn and apply problem-solving strategies in a structured way. Brief PST is typically delivered over 4–6 sessions and is suitable for delivery by a broad range of health professionals in various settings, including telephone, with high fidelity [20–23]. Pilot data suggests brief PST reduces diabetes distress and subthreshold depressive symptoms among adults with diabetes-related retinopathy [24].

The aim of the LISTEN (*L*ow-*I*ntensity mental health *S*upport via *T*elehealth *E*nabled *N*etwork) program is to provide evidence-based, early intervention to support adults with type 1 and type 2 diabetes experiencing diabetes distress. LISTEN will be facilitated by diabetes HPs (including credentialled diabetes educators, nurses, and dietitians) who will participate in an evidence-based training program. LISTEN uses brief PST as a framework for enhancing problem-solving skills, one of seven core diabetes self-management behaviours [25]. We have demonstrated the feasibility and acceptability of training diabetes health professionals to facilitate LISTEN, and of delivering such a program to adults with diabetes [23]. Our research has also suggested potential benefits of the approach, which need confirmation in a fully powered trial.

Our aims are threefold: (1) to examine the effectiveness and (2) cost-effectiveness of LISTEN for reducing diabetes distress and improving general emotional well-being among adults with type 1 and type 2 diabetes, in a hybrid type I effectiveness-implementation trial. The third aim is to explore the barriers to, and facilitators of, the adoption, implementation and sustainability of LISTEN when facilitated by diabetes HPs via telehealth. We hypothesise that LISTEN will (1) decrease levels of diabetes distress significantly at 6 months, compared to usual care; and (2) be cost-effective, with an incremental cost-effectiveness ratio below the commonly used threshold of $50,000/quality-adjusted life year (QALY).

## Methods

### Design

This study uses a hybrid type I effectiveness-implementation trial design [26] to test the effectiveness of LISTEN in a two-arm pragmatic, individual-level randomised controlled trial (RCT). We will simultaneously gather information on any barriers to, and facilitators of, implementation [26]. LISTEN (intervention) will be compared to usual care (comparison group), in terms of its impact on diabetes distress at 8 weeks (T1) and 6 months (T2) post-randomisation. To assess the cost-effectiveness of LISTEN, a within-trial economic evaluation will be conducted. Implementation will be examined using mixed-methods, and in accordance with the RE-AIM (Reach, Effectiveness, Adoption, Implementation and Maintenance) framework [27]. The Consolidated Framework for Implementation Research (CFIR) will inform exploration of multi-level barriers to, and facilitators of implementation and sustainability — including identification of implementation strategies to maximise delivery.

**Fig. 1:**
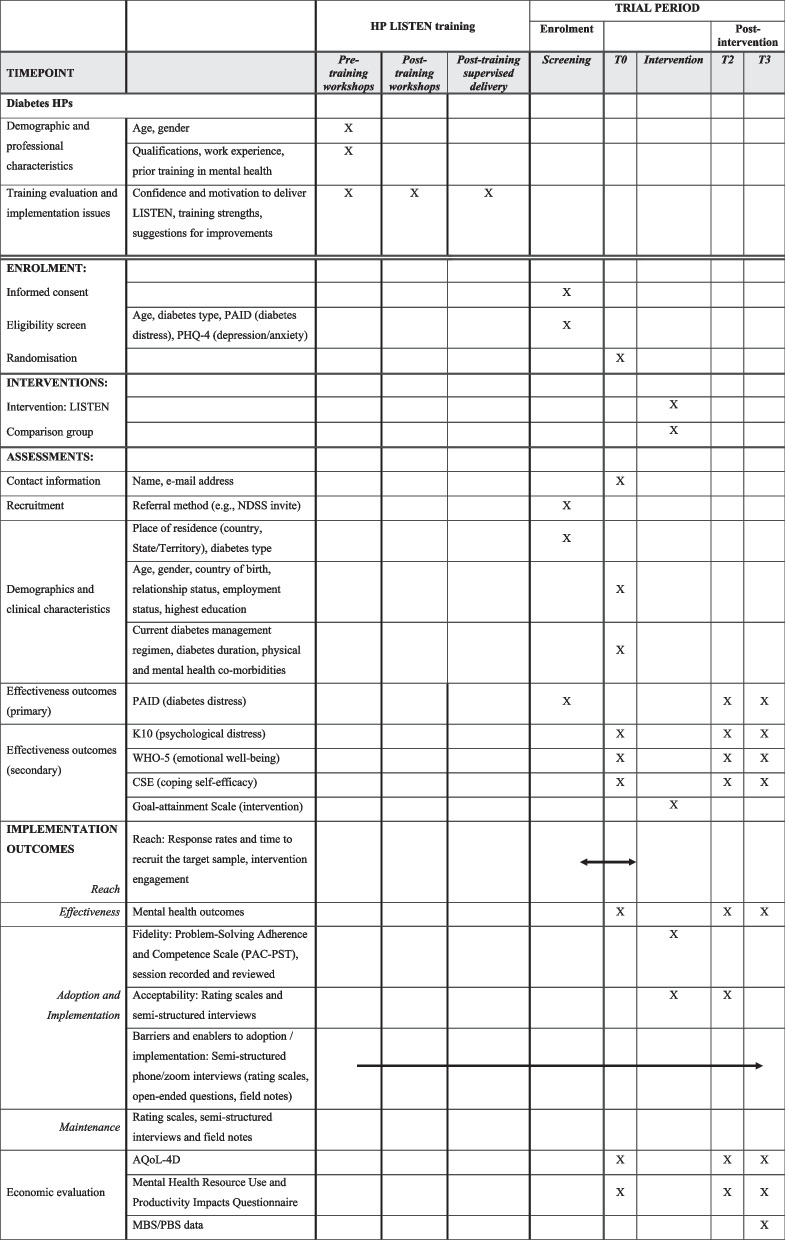
LISTEN trial schedule of enrolment, interventions, and assessments. HP, health professional; T0, baseline, T1, 8 weeks post-randomisation and allocation; T2, 6 months post-randomisation and allocation; PAID, Problem Areas in Diabetes Scale; PHQ-4, Patient Health Questionnaire-4; K10, Kessler Psychological Distress Scale; WHO-5, The World Health Organization-Five Well-Being Index; CSE, Coping Self Efficacy Scale; AQoL-4D, The Assessment of Quality of Life four-dimension instrument; MBS, Medicare Benefit Schedule, PBS, Pharmaceutical Benefits Scheme data

**Fig. 2:**
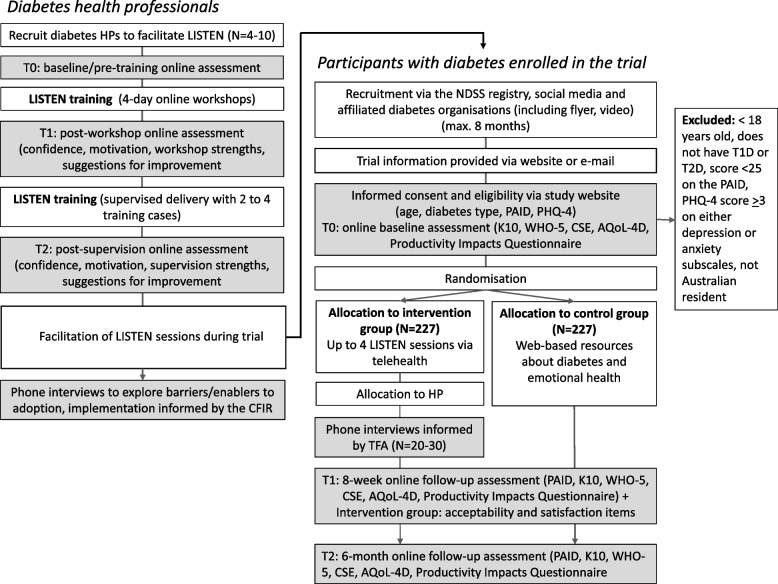
Flow of participants in the LISTEN study. HP, health professional; CFIR, Consolidated Framework for Implementation Research; NDSS, National Diabetes Services Scheme; PAID, Problem Areas in Diabetes Scale; PHQ-4, Patient Health Questionnaire-4; K10, Kessler Psychological Distress Scale; WHO-5, The World Health Organization-Five Well-Being Index; CSE, Coping Self Efficacy Scale; AQoL-4D, The Assessment of Quality of Life four-dimension instrument; TFA, Theoretical Framework of Acceptability

The protocol has been prepared according to the Standard Protocol Items: Recommendations for Intervention Trials (SPIRIT) statement. See Fig. 1 for schedule of enrolment, interventions, and assessments, as per the SPIRIT statement. Figure 2 shows the study flow. The trial will be reported in accordance with CONSORT guidelines for RCTs. The project board will meet quarterly to monitor the project timeline, risks, and quality assurance processes.

### Effectiveness trial

#### Participants and recruitment

Potential participants will be enrolled if they meet the inclusion criteria: adult aged 18 to 75 years; residing in Australia; self-reported diagnosis of type 1 or type 2 diabetes; at least mild diabetes distress (score ≥ 25 on the Problem Areas in Diabetes (PAID) scale (or a score of ≥ 2 (moderate problem) on three or more PAID items). LISTEN aims to provide early intervention to participants experiencing mild symptoms of depression and/or anxiety. Therefore, those with moderate-to-severe depression and/or anxiety symptoms (as indicated by a score ≥ 3 on either the depression or anxiety subscales of the four-item Patient Health Questionnaire (PHQ-4) will be excluded. Those with a score ≥ 3 will be referred to appropriate mental health services. A summary of the mental health inclusion criteria and referral pathways is in Supplementary File 1.

Participant recruitment will be primarily via e-mail invitation to adults with diabetes registered with the National Diabetes Services Scheme (NDSS). The NDSS is an Australian Government initiative, administered by Diabetes Australia, providing access to services, information and subsidised diabetes products (e.g. glucose monitoring supplies and insulin pump consumables). The NDSS register includes over 1.4 million Australians with diabetes and is considered reasonably accurate and up-to-date [28]. The NDSS will email invitations directly to potential participants on behalf of the project team. The research team will not have access to personal details unless potential participants make contact, or enrol in the study. E-mail invitations will be staggered over a 6–8-month recruitment period.

In addition, the study will be advertised on the Australian Centre for Behavioural Research in Diabetes (ACBRD) website, e-newsletter/blogs and social media (Twitter, Facebook) via the researchers’ affiliated professional accounts (e.g. Deakin University, ACBRD). National and state-based diabetes organisations (e.g. Diabetes Australia and Diabetes Victoria) will be encouraged to promote the study through similar methods.

#### Consent and procedure

The schedule of enrolment, intervention and assessment is detailed in Fig. 1. Study recruitment will be open for a maximum of 8 months (Fig. 2) or until the sample size (enrolled) is reached. Participation (from study entry to exit) will be for a duration of 6 months.

People with diabetes who are interested in participating will be directed to the study website to access the Plain Language Statement and provide informed consent. After providing consent, they will complete screening questions. Eligibility will be determined automatically based on responses. Those eligible will complete an online baseline assessment (T0) (hosted by Qualtrics™) and, will be randomly allocated to one of two study arms. Those who are ineligible will be informed immediately using an autogenerated message and provided with links to resources for mental health support.

Participants will be sent an email and SMS invitation with a link to the online follow-up assessment at 8 weeks (T1) and 6 months (T2) post-randomisation and allocation to the intervention or comparison group. The 8-week survey will be available for completion for 2 weeks. At the end of the first week, an email and SMS reminder will be sent to those who have not completed the survey. The 6-month survey will be available for completion for 3 weeks, with two reminders sent to non-responders at one and 2 weeks.

Participants will be e-mailed a $30 voucher on completion of the study (i.e. complete baseline, 8-week and 6-month follow-up assessments) as a token of appreciation, and to aid recruitment and retention.

#### Randomisation

Participants will be randomised (1:1 ratio) to either the intervention (LISTEN) or comparison group (usual care), via central randomisation by computer. The allocation will be fully concealed from the research team including the project manager (RG) and the trial statistician (BH). Upon randomisation, participants will be notified of their group allocation via e-mail by a research assistant independent of the investigator team. Randomisation will be conducted in random permuted blocks of participants, stratified by diabetes type, gender and age (< 60; ≥ 60 years old), in order to minimise imbalances in the group allocation overall and on the specific strata. Once they are allocated to an available HP, participants randomised to the intervention arm will receive a call from the facilitating HP within 1 week (approx.) to arrange sessions. The research team (except SG who will be conducting quality assurance) will remain blinded. Any violation of blinding will be recorded and reported with the main findings.

### LISTEN training for diabetes health professionals

HPs will be recruited via Diabetes Australia. A position description will be circulated internally as a first step. HPs will be volunteers and will complete a consent form prior to taking part in any aspect of the study. Eligibility criteria include Credentialled Diabetes Educator (or working towards credentialling) and Registered Nurse, Division 1 or Accredited Practising Dietitian; a minimum 12-month experience in Diabetes Education or working with people with diabetes in a community setting; an interest and motivation in supporting people with the emotional aspects of diabetes management; and experience providing structured education programs to consumers.

The HPs facilitating LISTEN will participate in comprehensive manualised training including (1) a 4-day online workshop and (2) supervised delivery of training cases. Following completion of the workshops, HPs will be allocated a minimum of two and a maximum of four training cases. Training cases are adults with diabetes who meet the inclusion criteria for the trial but who volunteer to be a ‘training case’. Sessions will be recorded and reviewed by a psychologist (SG) using the Problem-Solving Treatment Adherence and Competence Scale (PST-PAC) [30]. The PST-PAC examines fidelity to technical skills, adherence to the problem-solving steps, process tasks, communication and interpersonal effectiveness, and global competence based on the complexity of the client presentation. Fidelity is rated from 0 (*not completed*) to 5 (*well above standard*). Each session will be reviewed, and structured feedback provided to the HP during a weekly 1-h supervision session with SG, as well as unstructured discussions to promote micro-skills in session facilitation. To progress to the trial, HPs will be required to have completed ≥ 2 training cases, and achieved an overall PST-PAC score of ≥ 3 (*satisfactory*) for three consecutively-rated sessions, in accordance with an established PST training program [31].

### Intervention

**Fig. 3:**
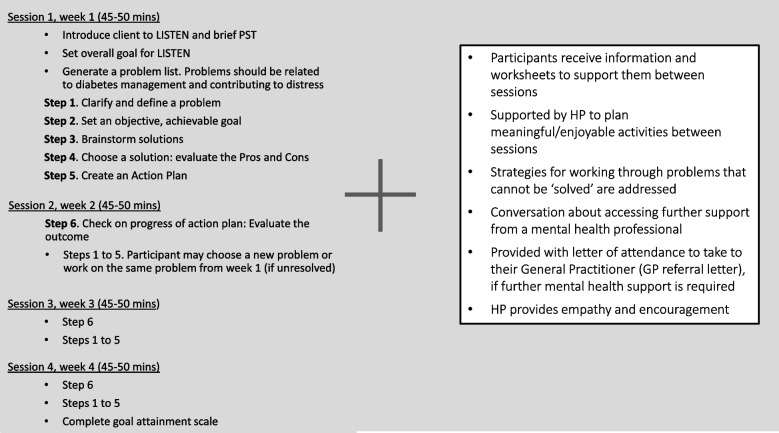
The LISTEN intervention

The intervention group will receive up to four LISTEN sessions (45–60 min each), facilitated by a diabetes HP via telehealth. Sessions will be offered weekly to allow participants sufficient time to implement their action plan (*homework tasks*) between sessions. The core of LISTEN provides participants with a 6-step approach to addressing problems related to their diabetes that are contributing to their distress (see Fig. 3). Participants receive information and worksheets to support them between sessions to plan meaningful and enjoyable activities. Where appropriate, HPs will provide participants with a letter of attendance to take to their General Practitioner (GP referral letter) to help initiate a conversation about accessing further support from a mental health professional.

### Comparison group

The comparison group will continue with their usual care and will receive a weblink to a freely available NDSS online factsheet about diabetes distress (https://www.ndss.com.au/wp-content/uploads/fact-sheets/fact-sheet-diabetes-distress.pdf), as well as links to general mental health support (e.g. Beyond Blue). They will also receive a general letter if they would like to discuss their emotional well-being with their GP.

During the trial, regardless of trial arm allocation, participants will not be asked to change their medications or diabetes management plan.

### Primary and secondary outcomes

All outcomes will be assessed pre-randomisation at baseline (T0), and post-randomisation at 8 weeks (T1) and 6 months (T2; primary endpoint).

#### Primary outcome

The primary outcome is the between-group difference in diabetes distress at T2. Diabetes distress will be assessed using the 20-item Problem Area in Diabetes (PAID) scale. Participants indicate which of the 20 diabetes issues are currently a problem for them, ranging from (0) ‘*not a problem*’ to (4) ‘*a serious problem*’. Item responses are summed and transformed to generate a scale score (0–100), with scores ≥ 40 indicative of severe diabetes distress. The PAID scale is valid, reliable and sensitive to change [32].

#### Secondary outcomes

General psychological distress will be assessed using the Kessler Psychological Distress Scale (K10). The scale contains 10 items about emotional states, rated on a five‐point scale, ranging from ‘*none of the time*’ to ‘*all of the time*’. The K10 score ranges from 10 to 50, with a higher score indicating greater psychological distress [33].

General emotional well-being will be assessed using the World Health Organization 5-item Well-being Index (WHO-5) [34]. Participants indicate symptom frequency over the past 2 weeks (0 = ‘*at no time*’ to 5 = ‘*all of the time*’). Item scores are summed and standardised to form a total score from 0 to 100. Higher scores represent better general emotional well-being.

Perceived ability to cope effectively with life challenges will be assessed using the Coping Self-Efficacy Scale (CSES) [35], which includes 26 items, each rated on a 11-point Likert scale, ranging from 0 (‘*cannot do at all*’) to 10 (‘*certain can do*’). Items form three subscales: problem-focused coping (12 items), stopping unpleasant emotions and thoughts (9 items) and getting support from family and friends (5 items), with higher scores indicating greater CSE. The total CSES scale score ranges from 0 to 260.

Generic health-related quality of life will be assessed using the 12-item Four-Dimensional Assessment of Quality-of-Life Instrument (AQoL4D) [36]. The scoring algorithm provides a total utility score on a scale from 0 to 1, based on Australian preferences for specific health states represented within the response items.

Information about the use of mental healthcare resources and lost productivity due to mental health problems will be captured using a Mental Health Resource Use and Productivity Impacts Questionnaire [37, 38].

The intervention group will also be invited to complete the Goal Attainment Scale [39]. At the beginning of their first LISTEN session, participants will set a goal for what they would like to achieve from the sessions. This will be recorded by the participant (or facilitating HP) on the PST worksheet. At the conclusion of their final session, participants will rate the extent to which their individual goals are attained (− 2 ‘*much less than expected*’ to + 2 ‘*much more than expected*’).

Demographic variables, collected at the baseline assessment (T0), include gender, age, education, marital status, employment status, mental health history and clinical characteristics (e.g. duration of diabetes, current treatment).

### Implementation outcomes

During the HP training and the RCT, we will use mixed methods to gather data on factors anticipated to affect the implementation of LISTEN, guided by the RE-AIM framework [40]. RE-AIM provides a structure for assessing health interventions beyond efficacy, with a focus on translatability into real-world settings.

#### Reach

Response rates, number of eligible participants and time to recruit the target sample will be monitored via the online registration system (Qualtrics). To determine whether participants are representative of people with diabetes in Australia, we will examine response rates by diabetes type, gender, age group, and geographical location (i.e. regional/remote areas). The number of eligible participants engaging in at least one LISTEN session will be monitored by an audit of the study database.

#### Effectiveness

Intervention effectiveness will be evaluated using a RCT (described above).

#### Adoption and implementation

##### Intervention fidelity

We will audit a random selection (20%) of LISTEN session audio recordings, stratified by HP and session number (1–4), to assess HPs’ fidelity to the program content (i.e. PST steps) and therapeutic techniques. Sessions will be rated independently (using the PST-PAC) by two experienced researchers and fidelity scores discussed until consensus is reached.

##### LISTEN training evaluation

HPs will be invited to complete three brief online surveys to explore satisfaction with the LISTEN training program as well as changes in confidence and motivation to facilitate sessions. The surveys will be (1) on entry into the study (baseline/pre-training), (2) following the LISTEN workshops, and (3) following the completion of their supervised training. Acceptability and satisfaction ratings are guided by the PRECEDE–PROCEED Model [41] and adapted from a previous published evaluation of health professional training in brief PST [42].

##### Intervention acceptability

Acceptability of LISTEN to participants in the intervention group, and their suggestions for improving sessions, will be explored using: (1) rating scales and open-ended questions at 8-week follow-up (T1); and (2) via semi-structured interviews with a random sample of 20–30 participants who undertake ≥ 1 LISTEN session). The random sample will be stratified to include a range of participants (e.g. by diabetes type, age, gender, number of sessions completed). The interview schedule is informed by the Theoretical Framework of Acceptability (TFA) [43] and adapted from a published interview exploring the acceptability of brief PST [21].

##### Barriers to, and facilitators of, adoption and implementation

We will conduct semi-structured interviews with 5–15 key stakeholders (i.e. HPs who facilitate LISTEN during the trial, representatives from Diabetes Australia and Diabetes Victoria involved in the study) to explore barriers and facilitators encountered during the HP training, LISTEN delivery and implementation strategies tested during the trial (Table 1). The interview schedule is aligned with the five Consolidated Framework for Implementation Research (CFIR) [44] domains. This will be supplemented with field notes (e.g. strategies discussed during supervision sessions with HPs to enhance intervention facilitation, strategies to enhance participant engagement and completion of LISTEN).

**Table 1:** Implementation strategies^a^ tested in the LISTEN trial

Strategy	Target	Stage of LISTEN study	Description and dose	Intended effect
Training materials and worksheets	HPs	HP training/trial	Training materials and worksheets will be provided to HPs during the LISTEN workshops, refined for trial (as needed)	Enhance skills acquisition and facilitation of LISTEN
Supervision	HPs	HP training/trial	Training: weekly individual 1-h sessions Trial: monthly individual 30-min sessions + ad hoc group sessions. All supervision will be provided by a psychologist (SG)	Enhance skills acquisition and facilitation of LISTEN, provide support and encouragement, promote intervention fidelity
Between supervision communication	HPs	Trial	As needed. Via e-mail, meetings, newsletters	Monitor HP scheduling to ensure a steady flow of participants are allocated to the intervention; maintain HPs motivation as a LISTEN facilitator (e.g. feedback from participants on the
Recruitment and promotional materials	Participating adults with diabetes	Trial	Refinements will be made to the recruitment/promotional materials in response to incoming data. This includes language used in recruitment flyer, short video, LISTEN information leaflet, social media posts	Increase enrolment of eligible participants into the trial
Processes and procedures	Participating adults with diabetes			Enhance engagement

^a^Implementation strategies are described according to recommendations published by Proctor et al. (2013) [29]

### Maintenance

Using all data sources above, we will identify pragmatic strategies to ensure both high-fidelity program delivery and implementation sustainability. An implementation and sustainability plan will be developed by the end of the study.

### Economic evaluation

To assess, from a health sector and broader societal perspective, the value for money of LISTEN compared to usual care (comparison), a within-trial economic evaluation will be undertaken along with economic modelling.

Detailed costing of the LISTEN intervention will be undertaken using financial data and micro-costing methods. At each time point within the trial, participants will be asked to complete a brief resource use questionnaire to collect information about other healthcare resources used and lost productivity [37, 38]. Participants will also be asked for consent to access their Medicare Benefit Schedule (MBS) and Pharmaceutical Benefits Scheme (PBS) data containing administrative information (numbers and cost details) on visits to health care providers and prescription medication covering the same time frame of study participation.

The AQoL-4D utility values for each participant at each time point will be used to calculate QALYs [45] using the area under the curve method. The within-trial economic evaluation will measure and value any change in healthcare resource use and then compare any additional costs to additional QALYs through an incremental cost-effectiveness ratio (ICER). Bootstrapping will be used to determine confidence intervals for the ICER and construct an acceptability curve to determine the cost-effectiveness of the intervention against the commonly used willingness-to-pay threshold of $50,000/QALY [46]. Sensitivity analyses will be undertaken to evaluate the robustness of results with changes to costing or analytical assumptions.

Scale-up and implementation costs as well as longer-term cost-effectiveness will be estimated based on population-wide modelling techniques based on published epidemiological data.

### Sample size

Based on a brief PST randomised pilot study assessing diabetes distress at 6 months [24] and assuming an alpha of 5%, we estimate requiring a minimum total sample of *N* = 226 (n = 113 per arm). This will enable us to detect an effect size of 0.3 (Cohen’s d) for the primary outcome, diabetes distress, with 80% power. Allowing for a 50% attrition rate observed in a trial with a similar recruitment strategy [47], we aim to recruit a minimum of *N* = 454 participants. Based upon prior experience using the NDSS register for recruitment to a trial [48], with a response rate of 1%, we estimate that recruiting a sample of *N* = 454 participants will require invitations to a random sample of *N* = 45,400 NDSS registrants.

### Data management

Data from all three timepoints will be downloaded from Qualtrics and linked according to the participant ID. Identifiable information will be separated from study data and stored along with the participant ID number in a password-encrypted Excel spreadsheet. All data will be stored in a secure electronic file accessible only by the research team. In accordance with clinical trial regulations, data will be kept for a minimum of 15 years after study completion before being disposed by erasing of electronic files.

### Planned analyses

Analysis of the primary outcome will be conducted on an intention-to-treat (ITT) basis, using repeated measures analysis of variance (ANOVA), or restricted maximum likelihood mixed-effects models (REML) [49]. The null hypothesis, that there is no difference in the primary outcome between the intervention and comparison groups (at T2), will be assessed using *p*-values for the fixed-effects for the associated covariates in each model, with a *p*-value < 0.05 deemed statistically significant. If participants have missing assessments at T2, the analysis of variance will be replaced by a mixed model analysis using the REML. A per-protocol set (PPS) will include only those participants who engage with all aspects of the study protocol and complete a minimum of one LISTEN session (intervention) or access the web-based resource (comparison) to be used for a sensitivity analysis of the primary endpoint. Secondary outcomes, including psychological distress (K10), general emotional well-being (WHO-5) and coping self-efficacy (CSES) will be analysed as per the primary outcome.

Qualitative data from semi-structured interviews will be de-identified, transcribed verbatim using a professional transcriber and imported into NVivo. Thematic analyses [50] will be used to explore and identify key themes about participant’s experiences of the LISTEN intervention and barriers to, and facilitators of, adoption and implementation.

There are no plans to conduct an interim analysis of the outcomes, as per the statistical analysis plan. Due to the low-risk nature of the intervention, we do not anticipate any harm associated with the intervention for participants, and thus have no stopping rules.

### Ethics and dissemination

This trial has received ethical approval from Deakin University Human Research Ethics Committee (DUHREC; Ref: 2021–412). This study will be conducted in compliance with this protocol (Version 5, March 2023), which was registered (1 February 2022, last updated 26 April 2023) with the Australian New Zealand Clinical Trials Registry (ACTRN: ACTRN12622000168752). Any protocol changes will be communicated to the human research ethics committee, funder, and ACTRN. The protocol registration will be updated with any approved amendments, and protocol departures will be documented in any reports or manuscripts resulting from this study.

The study findings will be disseminated to academic audiences, via presentation at scientific meetings, and publication in peer-reviewed journals. For non-academic audiences, including participants, a lay summary will be published via a freely available blog on the research team’s website and disseminated via e-newsletter. Study findings will also be reported to the funding body. On the consent form, participants will be asked if they agree to use of their data should they choose to withdraw from the trial. Participants will also be asked for permission for the research team to share relevant anonymous data with researchers who want to conduct further analysis.

All intervention-related adverse events (SAE) reported spontaneously by a participant during sessions and phone interviews, or through routine inspection of data from online assessments will be reported to the DUHREC within 24 h and communicated to the project board. A data safety monitoring board has not convened because the intervention is considered low risk.

### Stakeholder consultation

We have consulted with key stakeholders (adults with type 1 and type 2, diabetes HPs and representatives from diabetes organisations) to refine the study methodology, including the LISTEN intervention and LISTEN training of diabetes HPs. Further input will be sought from people with diabetes throughout the study; their roles will include advising on recruitment materials, making recommendations for implementation and scaling, and providing input into further refinements to the delivery of LISTEN and HP LISTEN training.

## Discussion

In Australia and internationally, there are a lack of real-world services that provide early intervention to enhance positive coping with the ongoing burden of living with diabetes. LISTEN aims to upskill diabetes health professionals to provide such early intervention, using structured, evidence-based, problem-solving skills and strategies, to meet the needs and preferences of people with diabetes. This trial will generate robust clinical outcomes data of the effectiveness and cost-effectiveness of LISTEN. Furthermore, our process evaluation will yield data on the barriers to, and facilitators of, the implementation and sustainability of LISTEN in practice, including evidence-based, pragmatic strategies to ensure high fidelity and sustainable program delivery. The findings will inform the implementation of LISTEN as a sustainable real-world service, designed to have an immediate and lasting positive impact on the emotional and mental health of Australians with diabetes.

## Trial status

The study protocol was registered before inclusion of the first participant on https://www.anzctr.org.au. Recruitment for this trial commenced in August 2022 and will continue until the target sample size is reached, which is anticipated in April 2023. We plan to complete the collection of 6-month follow-up data by October 2023.

The study protocol date: Initial approval December 2021; Current version 5, approved April 26, 2023.

## Supplementary Information

**Additional file 1.**

**Additional file 2.** Reporting checklist for protocol of a clinical trial.

## Data Availability

The research team members at the Australian Centre for Behavioural Research in Diabetes/Deakin University will have access to the trial dataset that is deidentified once the trial is completed. Data are available upon reasonable request. The final dataset will be available for researchers who are interested in the related topics after the research team has disseminated the main findings of the research aims. Permission from the lead investigator is required for any publications and dissemination effort.
