# Supplementary material for: Low-Intensity mental health Support via a Telehealth Enabled Network for adults with diabetes (LISTEN): protocol for a hybrid type 1 effectiveness implementation trial
Source: Trials. 2023 May 23;24:350. doi: 10.1186/s13063-023-07338-5 (PMC10204211; doi:10.1186/s13063-023-07338-5)
Supplement: Supplementary file 1 — Additional file 1. [file 13063_2023_7338_MOESM1_ESM.docx]

**Supplementary File 1.**

**Table 1**. Mental health inclusion and exclusion criteria for LISTEN trial

| **Identified level of distress** | **Participants’ score on:** | | **Eligible** |
| --- | --- | --- | --- |
|  | *PAID (0-100)^~^* | *PHQ-2, GAD-2^^^* |  |
| No/mild diabetes distress and no/mild general symptoms | Total <25 | <3 on either | No^a^ |
| Mild diabetes distress and no/mild general symptoms | Total <25; and  >2 on >3 items^#^ | <3 on either | Yes |
| Moderate-to-severe diabetes distress but no/mild general symptoms | Total 25-75 | <3 on either | Yes |
| Severe diabetes distress but no/mild depressive or anxiety symptoms | Total >75 | <3 on either | Yes |
| Severe diabetes distress plus at least moderate depressive or anxiety symptoms | Total >75 | >3 on either | No^b^ |

^~^ Scores ≥40 indicate severe diabetes-related distress and warrant further exploration and discussion with the person with diabetes.

^^^A score of 3 or greater on the 2-item depression or anxiety subscales represents a reasonable cut off point for identifying potential cases of depression/anxiety. A score of 3 or more is positive and should be further evaluated by PHQ-9 or a mental health referral should be made.

# Score of >2 on a PAID item = at least moderate problem; ^a.^ Offered referral to NDSS factsheet about diabetes distress; ^b.^ Provided with resources / referral to MH services and/or a referral to their GP.
